# Assessing White Spot Syndrome Virus (WSSV) and Acute Hepatopancreatic Necrosis Disease (AHPND) concurrent with *Vibrio* spp. in various *Penaeus monodon* aquaculture farms at southwestern region of Bangladesh

**DOI:** 10.1016/j.cirep.2024.200178

**Published:** 2024-10-28

**Authors:** Angkur Chowdhury, Chironjib Singha Samanta Chandan, Debasish Pandit, Basir Ahammad, Md. Habibur Rahman, Md. Imran Chowdhury, Rasel Mia, Shaila Akter, Md Zobayer Rahman, Bhaskar Chandra Majumdar

**Affiliations:** a Department of Fishery Biology and Genetics, Khulna Agricultural University, Khulna 9100, Bangladesh; b Department of Fish Biology and Genetics, Sylhet Agricultural University, Sylhet 3100, Bangladesh; c Department of Fishery Resources Conservation and Management, Khulna Agricultural University, Khulna 9100, Bangladesh; d Department of Fish Health Management, Khulna Agricultural University, Khulna 9100, Bangladesh; e Department of Oceanography, Khulna Agricultural University, Khulna 9100, Bangladesh; f Fisheries & Marine Resource Technology Discipline, Khulna University, Khulna 9100, Bangladesh; g Department of Aquatic Resource Management, Sylhet Agricultural University, Sylhet 3100, Bangladesh; h Department of Fish Health Management, Sylhet Agricultural University, Sylhet 3100, Bangladesh; i Department of Fisheries Technology and Quality Control, Khulna Agricultural University, Khulna 9100, Bangladesh

**Keywords:** *Peneaus monodon*, *Vibrio* spp., WSSV, AHPND, Aquaculture systems

## Abstract

This study was conducted to assess the prevalence of White Spot Syndrome Virus (WSSV) and Acute Hepatopancreatic Necrosis Disease (AHPND) in giant tiger shrimp (*Penaeus monodon* Fabricius, 1798) aquaculture systems (extensive, improved-extensive, and semi-intensive) in the southwestern region of Bangladesh. This research employed real-time polymerase chain reaction (RT-PCR) to quantification cycle (C_q_) values for identification of WSSV and AHPND in shrimp samples and *Vibrio* spp. was identified through biochemical tests on water and soil samples (*n* = 72 for each) from different aquaculture systems. The results of the study indicated significant difference (*p* < 0.05) in nitrate and ammonia levels across the various aquaculture systems, highlighting the critical need for rigorous water quality monitoring and management. Extensive aquaculture exhibited the highest prevalence of WSSV (40 %) and AHPND (33 %) positive samples, whereas semi-intensive aquaculture had the lowest prevalence, with 22 % WSSV-positive and 20 % AHPND-positive samples. Biochemical analysis identified three *Vibrio* spp. such as *V. cholerae, V. parahaemolyticus,* and *V. vulnificus* - with significantly higher concentrations in extensive aquaculture compared to semi-intensive. Notably, *V. vulnificus* was more abundant (0.9 × 10^3^ cfu/ml) in the soil of extensive aquaculture, while it was less prevalent in water. In conclusion, this study highlighted that the risk of WSSV and AHPND was greater in extensive aquaculture due to inadequate management.

## Introduction

In Bangladesh, the aquaculture industry plays a decisive role in ensuring a reliable supply of fish while also generating employment opportunities and profits for millions of individuals [[1], [2], [3], [4]]. To support food security and alleviate poverty in developing countries, it is crucial to optimize the utilization of resources in aquaculture to achieve the highest possible productivity [[5], [6]]. The prawn and shrimp production of Bangladesh in financial year 2022–23 was 3,13,984 MT (metric ton), with the Khulna division contributing the highest with 274,357 MT, which is about 87.38 % of the total production [7]. While aquaculture production and productivity in Bangladesh have been increasing steadily, the existing variability in productivity levels among individual farms indicates the presence of aquaculture production risks [[8], [9], [10]]. The increasing scope and variety of aquaculture methods in Bangladesh have led to the emergence of disease as a critical obstacle to maximizing fish production [11].

The rapid growth and intensification of shrimp farming have resulted in a corresponding rise in the incidence of diseases. This has precipitated a crisis within the industry, which can be attributed primarily to the heightened potency of pathogens such as *Vibrio* spp., as well as the prevalence of white spot viruses [12]. *Vibrio* spp. is salt-loving, gram-negative prominent aquatic bacteria that can survive with or without oxygen [13]. The genus *Vibrio* consists of over 130 species, with a minimum of 14 species being pathogenic in nature [14]. A well-known arthropod disease, namely Acute Hepatopancreatic Necrosis disease (AHPND), affects shrimps which was first reported in China in 2009 and the disease typically causes mortality rates of up to 100 % and was known to occur within the first 35 days after stocking shrimp fry of black tiger shrimp (*Penaeus monodon*) and whiteleg shrimp (*Litopenaeus vannamei*) [15]. Another lethal prawn and shrimp disease namely white spot disease is attributed to the white spot syndrome virus (WSSV), a large double-stranded DNA virus with a rod-shaped morphology, size ranges from 70 to 150 nm in diameter and 250 to 380 nm in length [16]. Also, the disease is characterized by a mortality rate of nearly 100 % within a period of 3 to 10 days [17].

Among various techniques available for virus detection such as observing clinical signs [18], wet mount microscopy [19,20], histology [21,22], conventional PCR (polymerase chain reaction), Multiplex PCR, nested PCR, RT-PCR (reverse transcription-polymerase chain reaction), and quantitative PCR [19,23,20,24]. Like this experiment, several studies documented in scientific literature have successfully employed RT-qPCR for fish disease detection purposes [[25], [26], [27], [28]]. For decade, RT-qPCR is widely used because of its access possibility, comparatively low cost, minimal required quantity of beginning points and extreme accuracy [29]. To measure the expression of genes, RT-qPCR methods aim to amplify a specific sequence of DNA, which represents a gene or other biological molecules, in a query sample. To start the amplification process, the sample is put in a well with a primer designed specifically for the DNA sequence that needs to be quantified. The quantity of sequence is estimated by counting the total number of cycles required to reach an earlier threshold set during the exponential amplification phase, when doubling of the product can be identified above background fluorescence [30]. These results are called quantification cycle (C_q_) values, which can also be referred to as threshold-cycle (C_t_) values. However, C_q_ is used here, in accordance with the uniform nomenclature proposed by Bustin et al. [31]. Through the comparison of the C_q_ values by analysis of specific DNA sequences can assess the quantity of DNA sequence in one compared to the other and highly recommended to account for systematic variation between samples [29]. Different species of *Vibrio* bacteria can be identified by their coloration, formation, appearance, and dimensions of colonies that develop on Thiosulfate Citrate Bile Salts Sucrose (TCBS) agar [32]. It is essential to implement biological techniques and use quarantine standards for farms at both national and community units to avoid and mitigate the effects of WSSV and AHPND [33]. The study is to assess the prevalence of WSSV and AHPND in extensive, improved-extensive, and semi-intensive aquaculture farms cultivating giant tiger shrimp (*P. monodon*) within the south-western region of Bangladesh. The study provides comprehensive insights into the health status of the *P. monodon* aquaculture farms in the south-western region of Bangladesh, contributing to the formulation of effective disease management strategies and sustainable aquaculture practices.

## Materials and methods

### Study area and experimental design

**Fig. 1:**
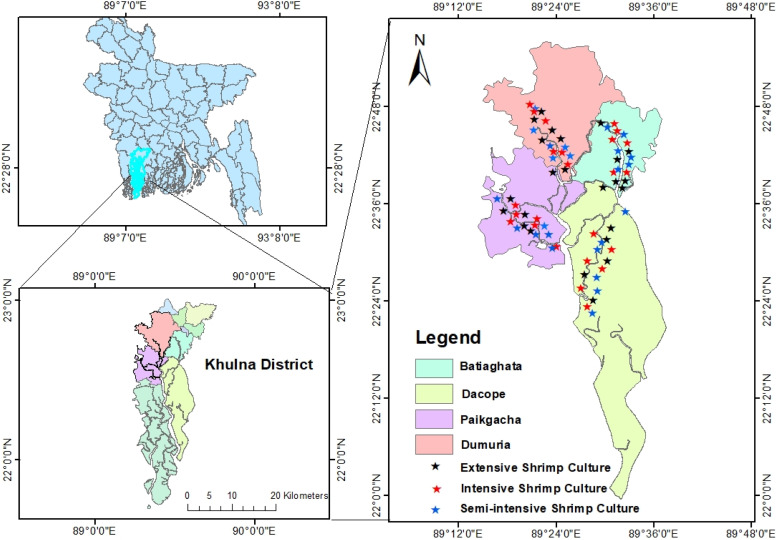
Map showing the study area of Dumuria, Paikgacha, Dacope and Batiaghata upazila of Khulna district (between 22°00′ to 22°48′ north latitude and 89°12′ to 89°48′ east longitude)*.*

The study was conducted in 72 aquaculture ponds distributed in four upazilas (sub-districts) (18 ponds per upazila) namely Dumuria, Paikgacha, Dacope and Batiaghata of Khulna district in the south-western region of Bangladesh (Fig. 1) where the production of *P. monodon* is higher than in other regions of the country. According to Ahmed et al. [34], the *P. monodon* aquaculture farms in the selected areas were classified into three categories such as extensive, improved-extensive, and semi-intensive aquaculture systems. Extensive aquaculture system is a conventional technique of farming where stocking was done by wild post-larvae of shrimp. In this system, home-made feeds, snail meat and organic fertilizer such as cow dung were supplied. Improved-extensive aquaculture system used more input than extensive but less than semi-intensive one. The stocking was done by both wild and hatchery produced post-larvae of shrimp where farm-made feeds as well as organic and inorganic fertilizers were applied. In the semi-intensive aquaculture system, farmers stocked only hatchery produced fry, supplied commercial pelleted feeds and lime, probiotics, urea and TSP (triple super phosphate) for pond management. The experiment was conducted in the duration of six-month from January to June 2023.

### Shrimp sample collection

Five shrimp samples (weight range: 3.00 to 80.54 g) were collected monthly from each selected pond in the study area covering three aquaculture systems. The shrimp samples were collected using cast net and kept sterile zipper beg to avoid cross-contamination. After collection, the samples were carefully placed in an ice box where the temperature was maintained at 2–4 °C and transported to the Fishtech Laboratory, Khulna for further processing and analysis.

### Water and soil sample collection

Water and soil samples were also collected at the same time for identification and quantification of *Vibrio* spp.. The water collection method was carried out by following Abioye et al. [35]. In briefly, water samples were taken by dipping a 250 ml sterilized falcon water bottle (Nalgene, United States), 20–25 cm down the water column from surface water. When the water bottle was still placed in the water column the bottle cap was added for sealing and marked with a writing marker including the date of the correction, and the name of the aquaculture pond for identification. The soil samples were collected using tiny PVC (Polyvinyl Chloride) pipe that had been air dried and cleaned with alcohol before the sample was transferred in a sterile easy-to-close zipper bag (FisherbrandTM Whirl-PakTM, United States). On the farm side, the water and soil samples were carefully placed in an ice box at 2–4 °C and these samples were finally stored at −40 °C freezer for subsequent analysis at the laboratory.

### Water quality parameter

Water quality was recorded monthly from each site covering all culture systems (*n* = 72) by using YSI ProDSS Multiparameter Digital Water Quality Meter (SKU-626,870–1, Yellow Springs, OH, USA) that captured various parameters associated with water quality. Water quality parameters including temperature, potential of hydrogen ion (pH), dissolved oxygen (DO), salinity, alkalinity, nitrate, and ammonia (NH_3_) were measured and documented.

### DNA extraction and quantifying pathogen-specific load using RT-PCR

Genomic DNA extraction was carried out using DNAzol reagent (Invitrogen) following the manufacturer's guidelines (Supplementary Fig. 1). Briefly, approximately 50 mg of gill tissue was homogenized in 1 ml of DNAzol, followed by centrifugation at 14,800×*g* for 10 min at 4 °C. The resulting supernatant was carefully transferred to a fresh tube. DNA precipitation was achieved by adding 0.5 ml of 100 % ethanol to the supernatant. The resulting DNA pellet was washed twice with 95 % ethanol through centrifugation and briefly air-dried. Subsequently, the dried DNA pellets were reconstituted in 100 µl of 8 M NaOH, incubated at 45 °C for 15 min, and then supplemented with 10 µl of Tris-EDTA buffer for storage at −20 °C. The detection of WSSV and AHPND was conducted using Real-time Polymerase Chain Reaction (RT-PCR) techniques. The CFX96 Touch Real-time PCR detection system (BioRad, Hrcules, CA, USA) was used to perform the PCR amplification. The DNA samples underwent quantitative real-time PCR assays, two additional primers, forward and reverse, were designed within the 739 bp WSSV DNA fragment and used in quantitative real-time PCR to produce a 45 bp amplicon. The recombinant plasmid functioned as an internal reference point for the estimation of WSSV, utilizing WSSV-specific primer pairs initially designed by Chakrabarty et al. [36] and the detection of AHPND was done utilizing the procedure described by Sirikharin et al. [37]. The protocol of PCR cycles condition includes primary denaturation at 94 °C for 5 min, second denaturation at 94 °C for 30 s and annealing at 60 °C for 1min. For amplification, 5 µL genomic DNA, 5.5 µL of nuclease-free water, 1 µL of specified forward and reverse primers (Macrogen, Inc., South Korea), and 12.5 µL of TB Green master mix (Takara Bio, Japan) were used in a 25 µL PCR mixture for amplification.

### Isolation of pathogenic microorganisms

After homogenizing the water and soil samples, 1 ml of each sample was transferred into enrichment broth in alkaline peptone water for the enrichment of *Vibrio* spp. These broths were then incubated at 37 °C for 6 h. After birth, 1 ml of the enriched broth was taken and subjected to 10-fold serial dilutions ranging from 10^−2^ to 10^−6^ in 9 ml of normal saline. From each of the dilution tubes corresponding to 10^−4^, 10^−5^, and 10^−6^, 0.1 ml of the diluted suspension was spread onto TCBS agar plates. After that, the agar plates were incubated for 24 h at 37 °C to promote colony growth. After incubation, characteristic colonies were identified and counted. This procedure was performed to detect and quantify *Vibrio* spp. present in the homogenized sample. To identify a particular pathogen, a number of biochemical tests were conducted, including gram stain, 1 % NaCl, slant, nitrate, gas, H_2_S, catalase test, oxidase test, indole, Methyl Red (MR), Voges Praskeur (VP), and citrate. Notably, this procedure was used to determine the presence and relative abundance of *Vibrio* spp. in the sample by utilizing selective media and colony-counting techniques.

### Statistical analysis

Statistical analysis and plotting of data were performed in R (version 4.2.3, [38]) using R-studio (version 2023.03.0, "Cherry Blossom"). Shapiro-Wilk normality test and Bartlett test of homogeneity of variance on bacterial colonies (cfu/ml) data and the C_q_ value data were run to evaluate the normality and homogeneity, respectively. Because the data were found normal and homogenous, a two-way ANOVA (followed by the HSD Tukey) as the *post hoc* test (*p* < 0.05) was performed to compare means of the average bacterial colonies (cfu/ml) and a one-way ANOVA (followed by the HSD Tukey) was performed to compare the C_q_ values for *P. monodon* samples and water quality parameters from extensive, improved-extensive and semi-intensive aquaculture systems. Several R-packages were used to perform analysis and create graphs including- “reshape2” [39] and “ggplot2” [40].

## Results

### Water quality parameters

Temperature, pH, salinity alkalinity and DO were not significantly differed among three aquaculture systems during the study period (Table 1). A lower DO concentration of 6.29 mg/l was found in the extensive aquaculture system, while the improved-extensive and semi-intensive systems were found to more DO i.e. 7.05 and 7.52 mg/l, respectively. The semi-intensive and improved-extensive aquaculture systems both had a nitrate level of 0.2 mg/l having significant difference (*p* < 0.05) with extensive aquaculture system that had a higher nitrate concentration (0.4 mg/l). Low NH_3_ concentrations were found in improved-extensive and semi-intensive aquaculture systems (0.03 and 0.01 mg/l, respectively), that significantly differed with extensive aquaculture system (0.6 mg/l).

**Table 1:** Overall water quality parameters (*n* = 72) in three different aquaculture systems of *P. monodon* throughout the study period. Different superscripts indicate significant differences (One-way ANOVA, *p* < 0.05).

Parameters	Extensive	Improved-extensive	Semi-intensive
Temperature ( °C)	27.6 ± 0.015^a^	27.5 ± 0.042^a^	27.6 ± 0.037^a^
pH	7.41±0.235^a^	7.30±0.028^a^	7.55±0.066^a^
DO (mg/l)	6.29±0.63^a^	7.05±0.24^a^	7.52±0.45^a^
Salinity (ppt)	15.3 ± 0.08^a^	16.8 ± 0.46^a^	16.7 ± 0.16^a^
Alkalinity (ppm)	160.26±7.08^a^	161.37±7.08^a^	161.12±5.08^a^
Nitrate (mg/l)	0.4 ± 0.00^a^	0.2 ± 0.00^b^	0.2 ± 0.00^b^
NH_3_ (mg/l)	0.60±0.03^a^	0.03±0.02^b^	0.01±0.03^b^

### Prevalence of WSSV and AHPND

The prevalence of WSSV varied across aquaculture systems. Extensive aquaculture system had a higher prevalence of WSSV (40 %) than improved-extensive and semi-intensive aquaculture systems having prevalence of 33 % and 22 %, respectively (Table 2). In case of AHPND, the extensive culture system also showed a higher prevalence (33 %) than improved-extensive (28 %) and semi-intensive (20 %) aquaculture systems (Table 3).

**Table 2:** Prevalence of WSSV at different sampling areas and months among different aquaculture systems (*n* = 30 samples/month/upazila).

Sampling sites	Months	Extensive	Percentage (%)	Improved-extensive	Percentage (%)	Semi-intensive	Percentage (%)
Dumuria	Jan – 23	10/30	33 %	8/30	27 %	5/30	17 %
	Feb – 23	12/30	40 %	10/30	33 %	7/30	23 %
	Mar – 23	16/30	53 %	10/30	33 %	8/30	27 %
	Apr – 23	12/30	40 %	7/30	23 %	8/30	27 %
	May – 23	11/30	37 %	9/30	30 %	6/30	20 %
	Jun – 23	10/30	33 %	11/30	37 %	9/30	30 %

Paikgacha	Jan – 23	15/30	50 %	11/30	37 %	7/30	23 %
	Feb – 23	16/30	53 %	12/30	40 %	8/30	27 %
	Mar – 23	13/30	43 %	10/30	33 %	8/30	27 %
	Apr – 23	12/30	40 %	9/30	30 %	8/30	27 %
	May – 23	11/30	37 %	9/30	30 %	4/30	13 %
	Jun – 23	13/30	43 %	9/30	30 %	5/30	17 %

Dacope	Jan – 23	12/30	40 %	11/30	37 %	4/30	13 %
	Feb – 23	11/30	37 %	12/30	40 %	4/30	13 %
	Mar – 23	13/30	43 %	10/30	33 %	5/30	17 %
	Apr – 23	12/30	40 %	9/30	30 %	6/30	20 %
	May – 23	11/30	37 %	9/30	30 %	6/30	20 %
	Jun – 23	10/30	33 %	11/30	37 %	7/30	23 %

Batiaghata	Jan – 23	11/30	37 %	10/30	33 %	8/30	27 %
	Feb – 23	12/30	40 %	9/30	30 %	3/30	10 %
	Mar – 23	11/30	37 %	9/30	30 %	9/30	30 %
	Apr – 23	10/30	33 %	11/30	37 %	10/30	33 %
	May – 23	14/30	47 %	11/30	37 %	6/30	20 %
	Jun – 23	12/30	40 %	10/30	33 %	7/30	23 %
	**Overall**	290/720	40 %	237/720	33 %	158/720	22 %

**Table 3:** Prevalence of AHPND at different sampling areas and months among different aquaculture systems (*n* = 30 samples/month/upazila).

Sampling sites	Months	Extensive	Percentage (%)	Improved-extensive	Percentage (%)	Semi-intensive	Percentage (%)
Dumuria	Jan – 23	12/30	40 %	8/30	27 %	7/30	23 %
	Feb – 23	7/30	23 %	10/30	33 %	6/30	20 %
	Mar – 23	11/30	37 %	5/30	17 %	8/30	27 %
	Apr – 23	9/30	30 %	9/30	30 %	6/30	20 %
	May – 23	11/30	37 %	8/30	27 %	7/30	23 %
	Jun – 23	10/30	33 %	10/30	33 %	5/30	17 %

Paikgacha	Jan – 23	8/30	27 %	9/30	30 %	4/30	13 %
	Feb – 23	11/30	37 %	8/30	27 %	4/30	13 %
	Mar – 23	7/30	23 %	9/30	30 %	4/30	13 %
	Apr – 23	11/30	37 %	10/30	33 %	7/30	23 %
	May – 23	10/30	33 %	11/30	37 %	6/30	20 %
	Jun – 23	13/30	43 %	10/30	33 %	8/30	27 %

Dacope	Jan – 23	9/30	30 %	8/30	27 %	8/30	27 %
	Feb – 23	10/30	33 %	8/30	27 %	6/30	20 %
	Mar – 23	12/30	40 %	9/30	30 %	6/30	20 %
	Apr – 23	10/30	33 %	10/30	33 %	6/30	20 %
	May – 23	9/30	30 %	8/30	27 %	7/30	23 %
	Jun – 23	10/30	33 %	11/30	37 %	5/30	17 %

Batiaghata	Jan – 23	12/30	40 %	7/30	23 %	5/30	17 %
	Feb – 23	9/30	30 %	9/30	30 %	7/30	23 %
	Mar – 23	12/30	40 %	6/30	20 %	6/30	20 %
	Apr – 23	8/30	27 %	5/30	17 %	5/30	17 %
	May – 23	11/30	37 %	9/30	30 %	6/30	20 %
	Jun – 23	6/30	20 %	8/30	27 %	6/30	20 %
	**Overall**	238/720	33 %	205/720	28 %	145/720	20 %

### Quantification cycle (C_q_)

**Fig. 2:**
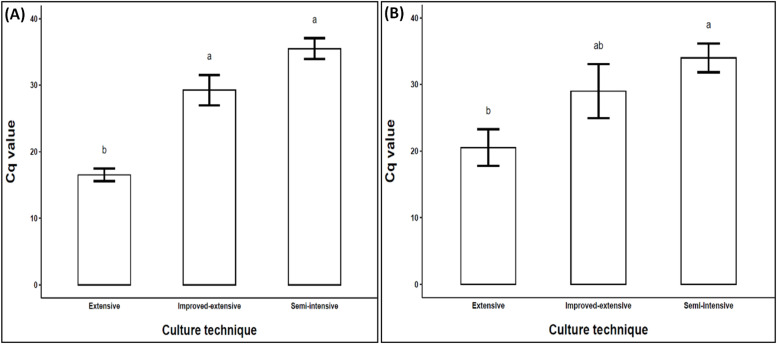
Threshold cycles (C_q_ value, mean ± standard error) of **(A)** White Spot Syndrome Virus (WSSV) and (B) Acute Hepatopancreatic Necrosis Disease (AHPND) in *P. monodon* muscle samples from extensive, improved-extensive and semi-intensive aquaculture systems. Different superscripts in the same figure indicate significant differences (*p* < 0.05, One-way ANOVA followed by HSD- Tukey as a post-hoc test).

The lowest C_q_ was observed in extensive aquaculture system (17) having significant difference (*p* < 0.05) with improved-extensive aquaculture system (30) and semi-intensive aquaculture system (37) for WSSV (Fig. 2A). In the case of AHPND, the C_q_ value (21) in extensive aquaculture system was significantly lower than semi-intensive aquaculture system (34), where no significant difference with improved-extensive aquaculture system (29) (Fig. 2B).

### Identification and quantification of *Vibrio* spp

Three *Vibrio* spp. such as *V. cholerae, V. parahemolyticus* and *V. vulnificus* were identified based on colony morphology and several biochemical tests. Table 4 entails an overview of the biochemical tests used to confirm specific pathogens based on colonies grown on TCB agar. Yellow colonies indicate *V. cholerae*, were negative on gram stain and gas producing, 1 % NaCl positive and positive on catalase and oxidase tests. The pathogen showed the ability to produce indole and utilize citrate while being negative in Methyl Red (MR) and Voges Praskeur (VP) assays. In contrast, purple or green colonies and bright yellow colonies associated with *Vibrio parahemolyticus* and *Vibrio vulnificus* showed similar characteristics except utilization citrate.

**Table 4:** Biochemical tests for the confirmation of specific pathogens of prepared colonies.

Colonies	Gram stain	1 % NaCl	Slant	Nitrate	Gas	H_2_S	Catalase test	Oxidase test	Indole	MR	VP	Citrate	Desired organism
TCB agar (2–4 mm) Yellow	–	+	A	+	–	–	+	+	+	–	–	+	*V. cholerae*
Purple or greenish	–	+	A	+	–	–	+	+	+	–	–	–	*V. parahemolyticus*
Bright yellow colony	–	+	A	+	–	–	+	+	+	–	–	–	*V. vulnificus*

*[A- Acidic Reaction; MR- Methyl Red; VP- Voges Praskeur; ±: Positive/Negative].

**Fig. 3:**
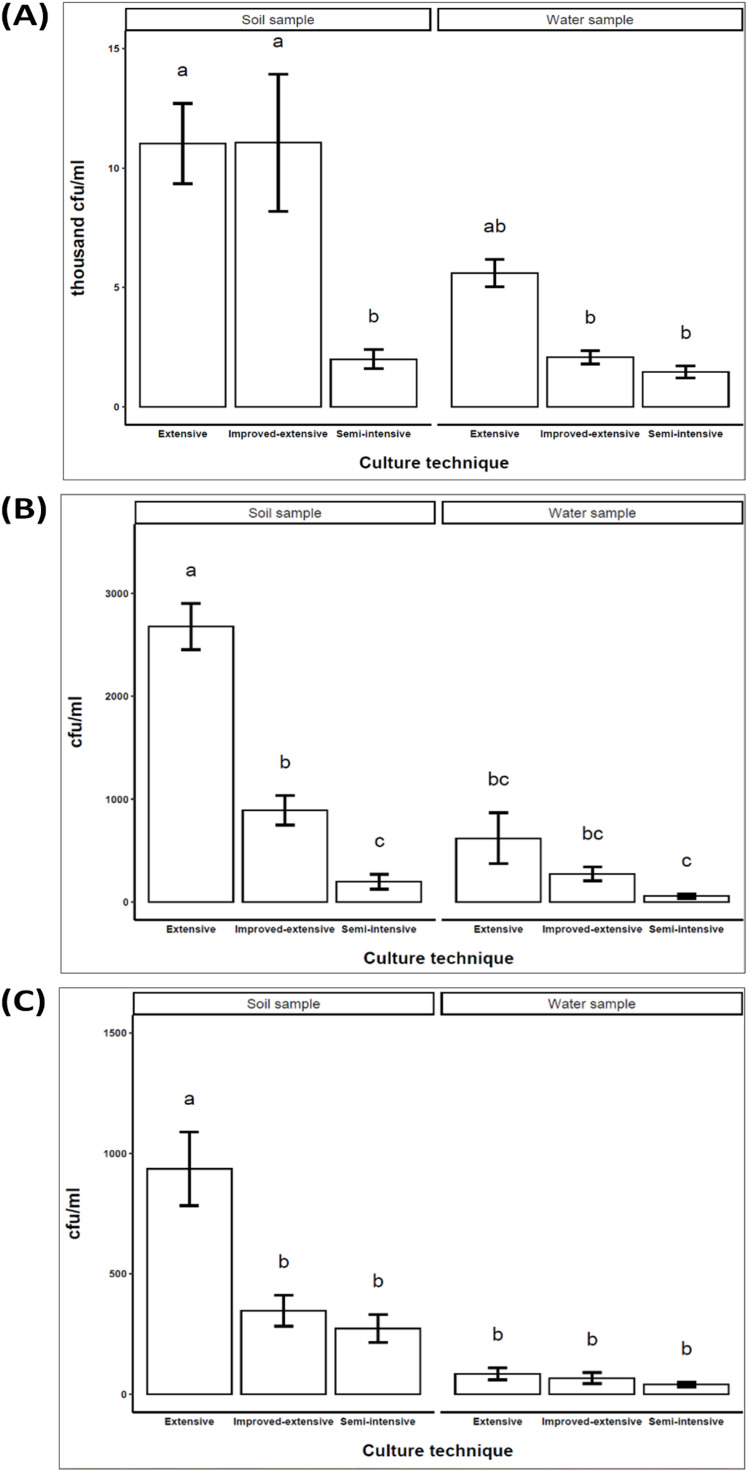
Colonies (mean ± standard error) of **(A)***V. cholerae* (thousand cfu/ml); (**B)***V. parahemolyticus* (cfu/ml); (C) *V. vulnificus* (cfu/ml) bacteria in soil and water sample from extensive, improved-extensive and semi-intensive aquaculture systems.

The number of *V. cholerae* colonies in the soil sample was significantly (*p* < 0.05) higher in the extensive (11.03×10^3^ thousand cfu/ml) and improved-extensive (11.07×10^3^ thousand cfu/ml) aquaculture system compared to the semi-intensive (2.0 × 10^3^ thousand cfu/ml, Fig. 3A). The presence of *V. parahemolyticus* in the soil sample was significantly (*p* < 0.05) higher in extensive (2.7 × 10^3^ cfu/ml) than in improved-extensive (0.9 × 10^3^ cfu/ml) and semi-intensive (0.2 × 10^3^ cfu/ml) aquaculture systems (Fig. 3B). The presence of *V. vulnificus* in the soil sample was significantly (*p* < 0.05) higher at extensive (0.9 × 10^3^ cfu/ml) than in improved-extensive (0.3 × 10^3^ cfu/ml) and semi-intensive (0.2 × 10^3^ cfu/ml) aquaculture systems (Fig. 3C). The colonies of all three *Vibrio.* spp. was found higher in the extensive compared to the semi-intensive aquaculture system. However, the colonies of *V. cholerae* and *V. parahemolyticus* from water samples were significantly higher in the extensive aquaculture system and lower in the semi-intensive aquaculture system (*p* < 0.05, Fig. 3). The colonies of the water sample from three aquaculture systems of *V. vulnificus* showed no significant differences.

## Discussion

Most of the water quality parameters in all aquaculture systems were within suitable range for shrimp culture and other aquatic lives [[41], [42], [43]]. The highest value of pH and DO was recorded in semi-intensive aquaculture system. On the other hand, improved-extensive aquaculture system showed the highest value of salinity and alkalinity providing valuable information for culture management and diseases control strategies. The extensive aquaculture system had significantly higher (*p* < 0.05) concentrations of nitrate and NH_3_ which could be harmful to aquatic life [44,41,42]. Microorganisms like WSSV and *Vibrio* spp. have consistently posed severe threats to sustainable shrimp farming [45]. For these lethal microorganisms, shrimp farms in Bangladesh often experience mass mortality of fry and adults, reduced production, and financial stress [34]. In the current study, the highest prevalence of WSSV (40 %) and AHPND (33 %) positive samples was found in extensive aquaculture system; the lowest prevalence was seen in semi-intensive aquaculture system, with 22 % of WSSV-positive samples and 20 % of AHPND-positive samples. Within the realm of diagnostic methodologies, PCR stands out for its remarkable sensitivity and specificity when it comes to identifying WSSV [46]. AHPND vulnerability were documented in *P. monodon* and *P. vannamei*, as noted by various sources [47,48]. Employing a similar approach, WSSV prevalence of 20 % in indigenous *P. monodon* brooders in Taiwan was documented by Kou et al. [49]. Likewise, in Thailand, a prevalence rate of 18 % was observed at the peak of occurrence between September and November, as assessed through the application of the PCR technique [50]. In the context of hatchery-reared post-larvae of *P. monodon*, instances of multiple infections involving WSSV alongside Hepatopancreatic Parvovirus (HPV), Monodon Baculovirus (MBV) had been documented [51,52]. The presence of WSSV has been detected not only in *P. monodon* from Andaman waters but also in other crustaceans like the mud crab, *Scylla serrata*, and the banana shrimp, *Fenneropenaeus merguiensis* [53,54]. Moreover, WSSV has demonstrated its capacity to infect freshwater crabs with significant virulence, leading to total mortality in case of infections in controlled environment [55]. Notably, various genotypes of WSSV have been identified within Indian shrimp farms, each exhibiting diverse levels of infectivity potential [40]. Recent investigations unveiled instances of WSSV infection among L. *vannamei* within semi-intensive culture setup in India [56]. Additionally, the occurrence of WSSV and MBV prevalence within the wild broodstock of *P. monodon* has also been documented [57]. According to Lai et al. [58], the most frequently documented indicator of AHPND in affected shrimp is the presence of a shrunken and pale hepatopancreas (HP). The initial stages of AHPND in shrimp are characterized by the manifestation of a pale to white HP, which aligns with the current experiment. This results from the depletion of pigments in the HP R-cells and is accompanied by the atrophy of the HP, potentially causing a reduction in the organ's size by 50 % or even more [59]. The highest C_q_ value in RT-PCR indicate lower amount of target nucleic acid and the lowest C_q_ value indicate higher amount of target nucleic acid [28]. This study showed that the higher C_q_ value for semi-intensive aquaculture system indicated lower amount of target nucleic acid of WSSV and AHPHD compered to improved-extensive and extensive aquaculture systems.

The presence and lethal effect of these *Vibrio* spp. have been documented globally [35,32,60]. Southeast Asian countries' seafood markets such as shrimp market, are often adulterated with pathogenic *Vibrio* spp. [61]. During this time, shrimp progressively deteriorate, impacting product quality [62]. To address these concerns, the present study was conducted to evaluate the pathogenic load in shrimp samples intended for export from different aquaculture systems- extensive, improved-extensive and semi-intensive, focusing on maintaining their quality standards. The number of *Vibrio* spp. colonies in water and soil samples were examined as indicators of the farm environment. According to biochemical test, three *Vibrio* spp. like *V. cholerae, V. parahaemolyticus* and *V. vulnificus* were shown to be substantially more prevalent in extensive aquaculture system as opposed to semi-intensive aquaculture system. Lima et al. [60] investigated *Vibrio* spp. in 14 shrimp farms in Northeastern Brazil and revealed higher bacterial indices in shrimp ponds (1.7 × 10^3^ to 1.3 × 10^4^ cfu/ml) compared to inlet water (1.4 × 10^3^ to 4.7 × 10^3^ cfu/ml). Therefore, the higher amount of *Vibrio* spp. may indicate water quality degradation and risk of disease outbreak. Likewise, semi-intensive shrimp farms have a lower risk of disease outbreaks than extensive farms. The improvement of farming characteristics reduces the disease risk of shrimp farms. Few studies detected *V. vulnificus* in shrimp culture ponds [63,62]. On the contrary, *V. parahaemolyticus* inhabits aquatic surroundings, encompassing elements such as sediments, plankton, and aquatic organisms [64]. As documented by Gopal et al. [63], the examination of water samples revealed a greater concentration of viable *Vibrio* spp. on the western coast (∼10^4^ cfu/ml) when compared with samples from the eastern coast (∼10^2^ cfu/ml). This finding corroborates earlier observations from other experiments [15,65]. Furthermore, Hossain et al. [66] determined that the total *Vibrio* counts ranged up to 2.5 × 10^3^ cfu/g and 60 cfu/g in samples from shrimp and Gher water, respectively. Joshi et al. [67] performed a similar study in a Thai shrimp farm that explained the examination of *V. parahaemolyticus* isolates. Overall, the nitrate and ammonia content were found higher in extensive ponds which may have facilitated the amplification of WSSV and *Vibrio* spp. It has been documented in several experiments that poor management practices can facilitate disease outbreaks in aquaculture farms and vice-versa [68,69]. Proper water quality management of farms and regular health monitoring can reduce the disease risk of *P. monodon* farms in south-western region of Bangladesh.

## Conclusion

In conclusion, the study revealed varying prevalence rates of WSSV and AHPND in *P. monodon* across different aquaculture systems in the south-western region of Bangladesh. The study emphasized the importance of maintaining stringent biosecurity protocols and enhancing water quality management to mitigate the spread of these virus and disease. Future experiments should be focused on exploring the health and water quality management techniques that can tackle viral diseases.

## CRediT authorship contribution statement

**Angkur Chowdhury:** Writing – review & editing, Writing – original draft, Visualization, Validation, Supervision, Software, Resources, Project administration, Methodology, Investigation, Formal analysis, Data curation, Conceptualization. **Chironjib Singha Samanta Chandan:** Writing – original draft, Project administration, Methodology, Investigation, Formal analysis, Data curation. **Debasish Pandit:** Project administration, Methodology, Investigation, Formal analysis, Data curation. **Basir Ahammad:** Writing – original draft, Project administration, Methodology, Investigation, Formal analysis, Data curation. **Md. Habibur Rahman:** Project administration, Methodology, Investigation, Formal analysis, Data curation. **Md. Imran Chowdhury:** Methodology, Investigation, Formal analysis, Data curation. **Rasel Mia:** Methodology, Investigation, Formal analysis, Data curation. **Shaila Akter:** Investigation, Formal analysis, Data curation. **Md Zobayer Rahman:** Investigation, Formal analysis, Data curation. **Bhaskar Chandra Majumdar:** Writing – original draft, Project administration, Methodology, Investigation, Formal analysis, Data curation.

## Declaration of competing interest

The authors declare that they have no conflict of interests.

## Data Availability

Data will be made available on request.

## Appendix Supplementary materials

Image, application 1
