# Supplementary material for: Assessing White Spot Syndrome Virus (WSSV) and Acute Hepatopancreatic Necrosis Disease (AHPND) concurrent with Vibrio spp. in various Penaeus monodon aquaculture farms at southwestern region of Bangladesh
Source: Comp Immunol Rep. 2024 Oct 28;7:200178. doi: 10.1016/j.cirep.2024.200178 (PMC11570757; doi:10.1016/j.cirep.2024.200178)
Supplement: Supplementary file 1 [file mmc1.docx]

**Supplementary Information:**


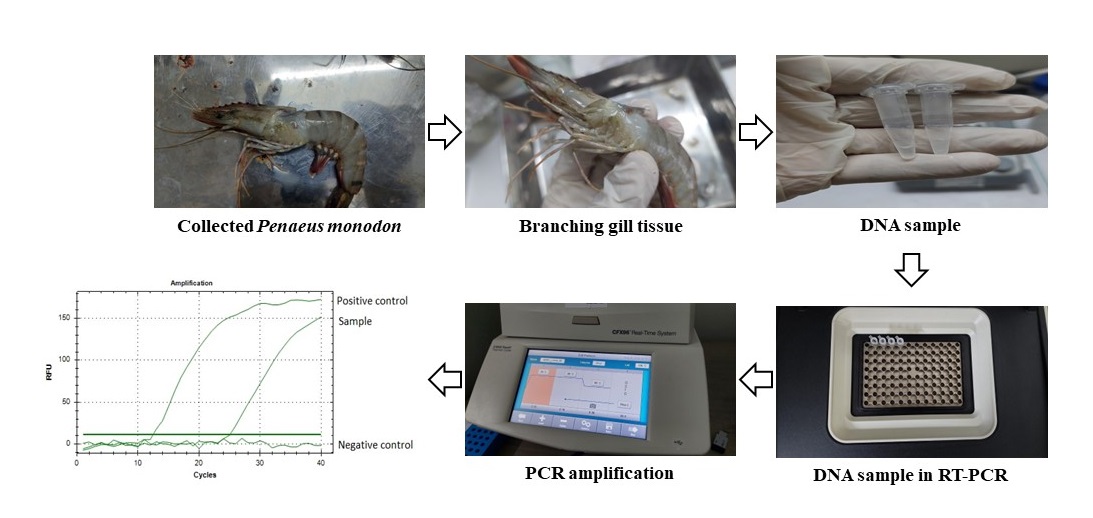


**Supplementary Fig. 1:** Extraction of DNA from branching gill tissue and RT-PCR amplification.
